# A case of dupilumab‐induced psoriasis‐like eruption treated with baricitinib

**DOI:** 10.1002/ski2.338

**Published:** 2024-01-16

**Authors:** Yuki Kinoshita, Hisayoshi Imanishi, Tomoko Oshimo, Daisuke Tsuruta

**Affiliations:** ^1^ Department of Dermatology Osaka Metropolitan University Graduate School of Medicine Osaka Japan

## Abstract

Dupilumab is a humanized monoclonal antibody against the interleukin (IL)‐4 receptor alpha chain that inhibits IL‐4 and IL‐13 signalling. It is one of the systemic treatments for patients with moderate‐to‐severe atopic dermatitis (AD), which provides favourable safety and efficacy. We report a case of psoriasis‐like eruption induced by dupilumab as an adverse effect in a patient with AD, immediately remitted after switching to baricitinib, which inhibits JAK1/2. Moreover, the atopic skin lesion also simultaneously went into remission upon baricitinib treatment. Baricitinib could be a favourable option for patients with AD who develop dupilumab‐induced psoriasis‐like eruption.

## Graphical abstract

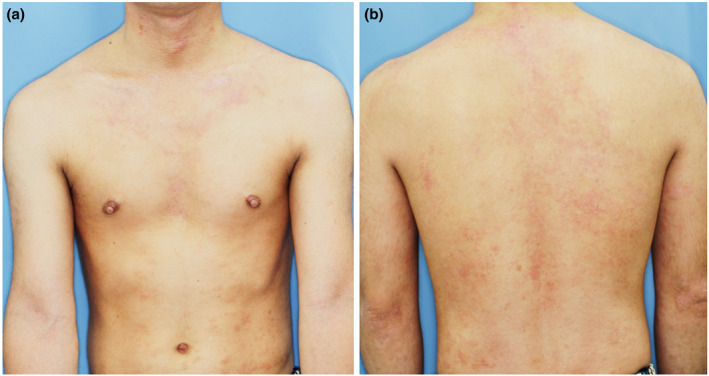

## 1 INTRODUCTION

Dupilumab is an interleukin (IL)‐4 receptor antagonist that inhibits IL‐4 and IL‐13 signalling.^1^ Since IL‐4 and IL‐13 are key mediators of T‐helper (Th) 2‐mediated inflammation, their blockade by dupilumab could promote a shift from Th2‐mediated inflammation to Th1/Th17 subsets, inducing psoriasis.^1^, ^2^ IL‐4 reportedly downregulates Th1 and Th17 cells, which are major inflammatory mediators in psoriasis, and can modulate dendritic cells, thereby suppressing IL‐23 production.^1^, ^2^ Herein, we report a case of dupilumab‐induced psoriasis‐like eruption, which immediately remitted after switching to baricitinib. The atopic skin lesion also simultaneously went into remission.

## 2 CASE

**FIGURE 1:**
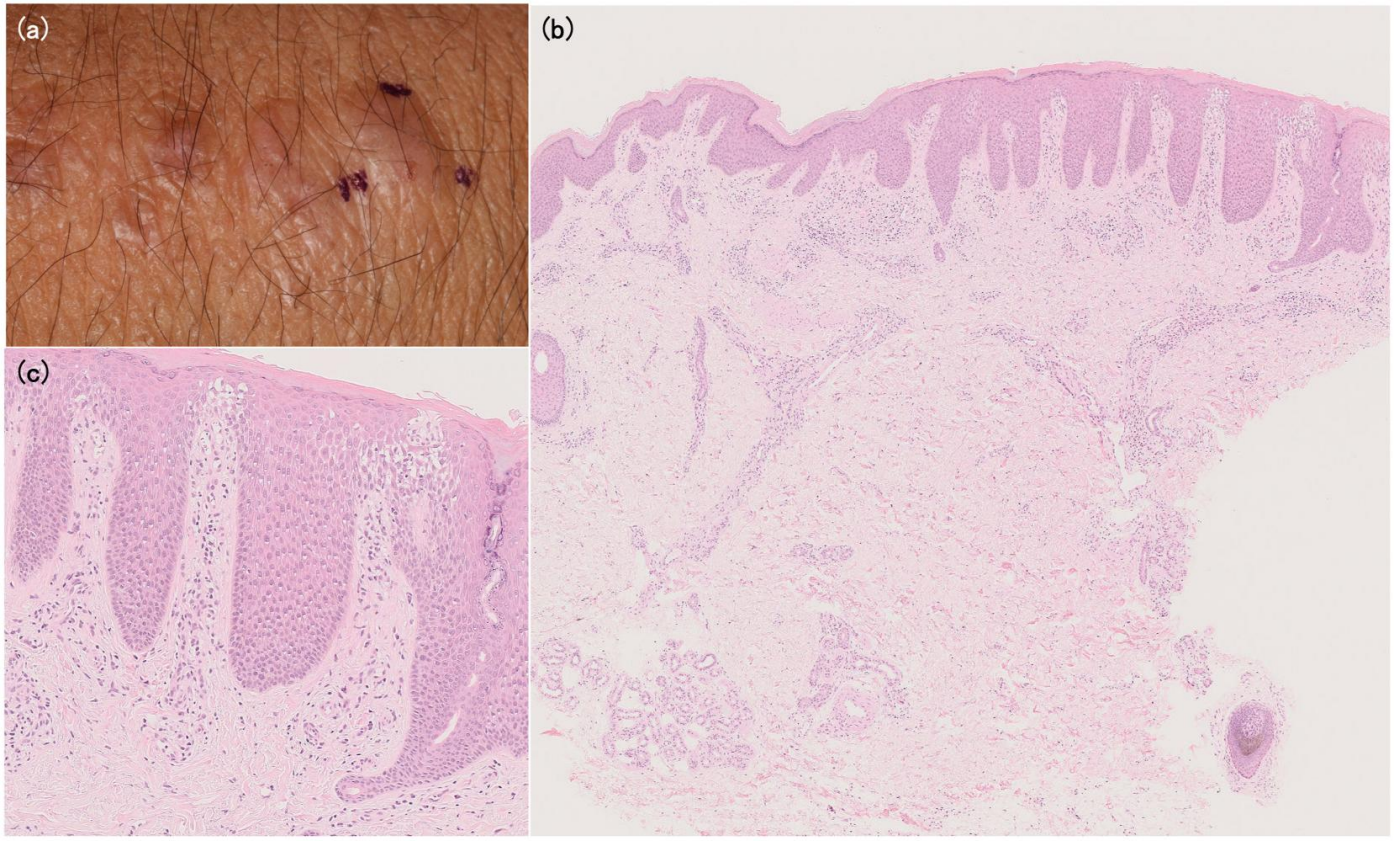
(a) Erythematous nodules on the dorsum of the hand at the first visit. (b, c) Haematoxylin–eosin staining of the biopsy specimen from the erythematous nodule showed psoriasiform dermatitis with infiltration of inflammation cells in the upper dermis, which was a finding consistent with atopic dermatitis. Original magnification ×100 (b) and ×400 (c).

**FIGURE 2:**
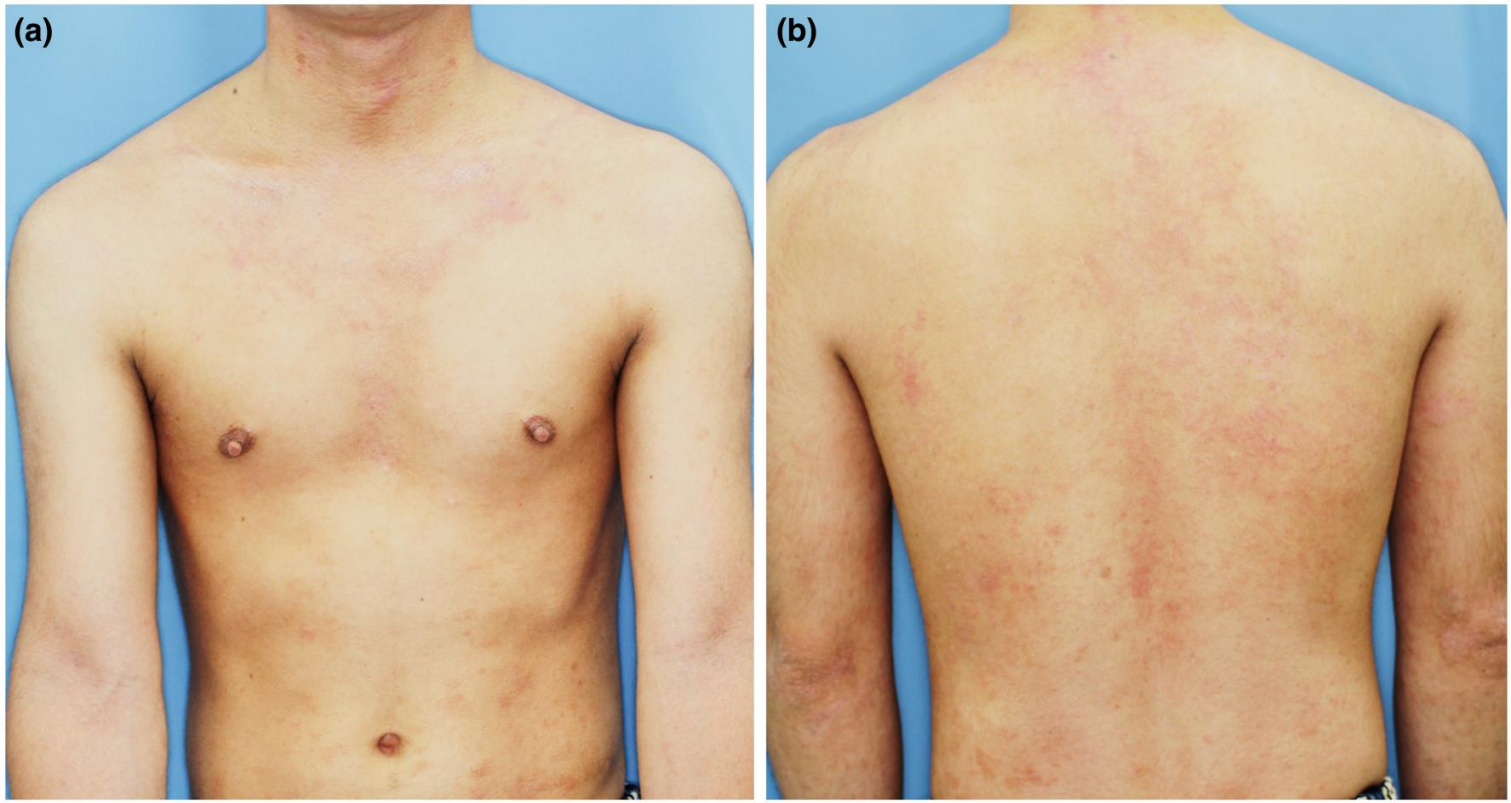
(a, b) Clinical evaluation of atopic dermatitis at the beginning of treatment with dupilumab. Erythema, erythematous papules, scales with lichenification, and excoriations were symmetrically present on the entire body, including the front of the trunk (a) and on the back (b).

**FIGURE 3:**
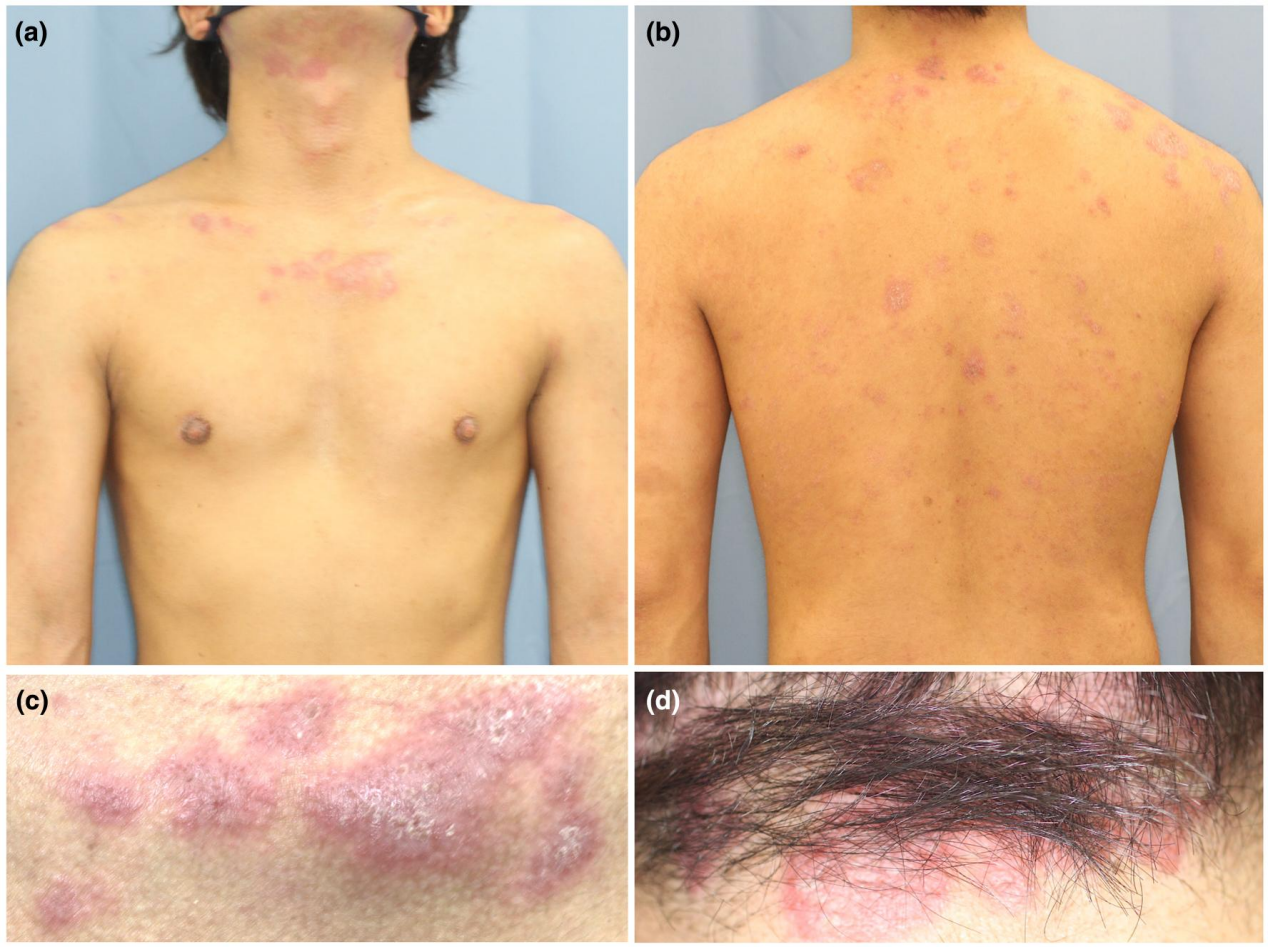
(a–d) Clinical evaluation of psoriasiform eruption that appeared 2 months after resuming dupilumab treatment. Multiple, bright red, sharply and well‐defined plaques with thick scales appeared on the whole body, including on the front of the trunk (a, c) and on the back (b, d).

A 31‐year‐old man presented with atopic dermatitis (AD) from childhood, associated with elevated total serum IgE. He had no personal or familial history of psoriasis vulgaris. Clinical examination revealed erythema, erythematous papules, and lichenification with scales and scratches, which were symmetrically present on his entire body (Eczema Area and Severity Index [EASI] = 39.8). Skin biopsy from the erythematous nodule on the dorsum of the hand revealed psoriasiform dermatitis with infiltration of inflammation cells in the upper dermis, which was a finding consistent with AD (Figure 1a–c). Based on the chronic clinical course, characteristic skin rash morphology and distribution, and histological findings, the patient was diagnosed with AD. His AD was poorly controlled, despite treatment with topical steroids of the strongest class, oral antihistamines, prednisolone, and cyclosporine, and narrowband ultraviolet B phototherapy. Therefore, at 1 year after the initial visit, we started dupilumab treatment (EASI = 20.9, Figure 2a,b). The skin lesion became milder but did not respond as well as the patient had hoped. Therefore, 2 years after the initial diagnosis, we followed the patient's request to discontinue the drug due to a lack of satisfaction with the cost. However, the skin manifestation was poorly controlled, and dupilumab treatment was resumed 3 years after the initial diagnosis. 8 weeks later, the patient developed multiple, bright red, sharply and well‐defined plaques with thick scales on the whole body (Psoriasis Area and Severity Index = 10.4, Figure 3a–d). Although we could not obtain the patient's consent to perform the skin biopsy, we diagnosed the skin lesion with dupilumab‐induced psoriasis‐like eruption based on the characteristic skin rash morphology. We discontinued dupilumab, and started oral prednisolone at 10 mg/day, tapered off for 2 weeks. Because the psoriasis‐like skin manifestations did not improve, we started baricitinib 4 mg/day 4 weeks after the psoriasis‐like eruption appearance. Around 80% of the red, well‐defined, scaly plaques, erythema, and red papules faded and pigmented 4 weeks after starting baricitinib and completely disappeared by the time of the visit 8 weeks later. The AD simultaneously became well‐controlled. Baricitinib was continued for 6 months, with no relapse of psoriasis‐like eruption and good control of AD, but was discontinued due to financial circumstances. Subsequently, only AD flared up, and baricitinib was restarted 1 year later. Four months after resumption of baricitinib, AD is well‐controlled and psoriasis‐like skin rash does not appear (Table 1).

**TABLE 1:** The timeline summarizing the patient's clinical course.

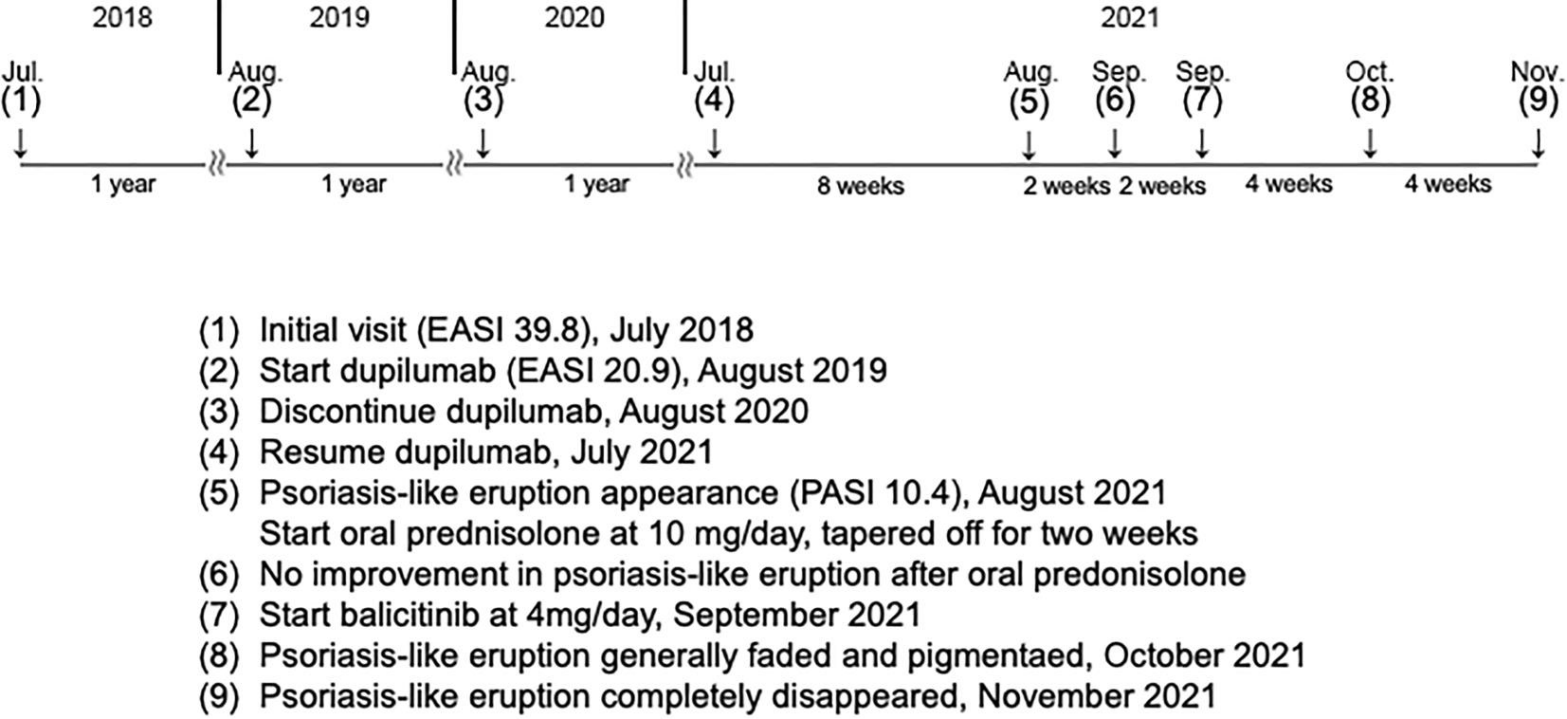

## 3 DISCUSSION

Ferrucci et al. reported a case of dupilumab‐induced psoriasiform eruption that resolved with oral methotrexate (MTX). The skin lesion flared up again upon starting upadacitinib (JAK1 inhibitor) treatment, and then disappeared again upon upadacitinib discontinuation and restarting of oral MTX.^3^ They mention that MTX is speculated to inhibit both JAK1 and JAK2, possibly providing wider inhibition of the inflammatory pathways responsible for both AD and psoriasis.^3^ They suggested that suboptimal inhibition of tyrosine kinase 2‐mediated cytokines (e.g. IL‐12 and IL‐23) may have led to psoriasiform eruption upon switching from MTX to upadacitinib.^3^ In our case, baricitinib also blocked the JAK2 cascade, which may have inhibited IL‐12 and IL‐23, yielding a rapid resolution of the psoriasis‐like eruption and subsequent remission. Ali et al. reported cases of twin dupilumab‐induced psoriasiform eruption that resolved with baricitinib, as in our case.^4^ In the paper, mRNA (RNA‐FISH) cytokines gene expression in the lesional skin biopsy specimens showed a significantly high concentration of IL‐17A. Blood tests revealed a high concentration of IgE and eosinophils, and cytokines detection in blood showed IL‐5, IL‐6 and IL‐17 in one patient; however, only IL‐5 in another patient. The authors supposed that the balance might shift towards Th1/Th17 predominance, and psoriasis develops by suppressing skewed Th2 activation in patients with AD although the heterogeneity exists.^4^

This is the rare report of psoriasis‐like eruption triggered by dupilumab and resolved by baricitinib treatment, which also yielded good control of AD. Baricitinib, a JAK1/2 inhibitor, could be a favourable option for patients with AD who develop dupilumab‐induced psoriasis‐like eruption.

## CONFLICT OF INTEREST STATEMENT

The authors declare no conflicts of interest.

## AUTHOR CONTRIBUTIONS

**Yuki Kinoshita**: Writing – original draft (lead); writing – review & editing (lead). **Hisayoshi Imanishi**: Supervision (lead); writing – review & editing (equal). **Tomoko Oshimo**: Supervision (supporting); writing – review & editing (supporting). **Daisuke Tsuruta**: Supervision (supporting); writing – review & editing (supporting).

## ETHICS STATEMENT

Not applicable.

## Data Availability

Research data are not shared.
